# Retreatment of Two Bioceramic Sealers Included Two Different Percentages of Calcium Silicate Using Two Endodontic File Systems: An *In Vitro* Study

**DOI:** 10.1055/s-0045-1808262

**Published:** 2025-05-07

**Authors:** Mohamad Alouda, Samar Akil, Ammar Eid, Filippo Cardinali, Hassan Achour, Youssef Haikel, Naji Kharouf

**Affiliations:** 1 Department of Endodontic and Conservative Dentistry, Damascus University, Damascus, Syrian Arab Republic; 2 Department of Endodontics and Operative Dentistry, Faculty of Dentistry, International University for Science and Technology (IUST), Ghabagheb, Syrian Arab Republic; 3 Private Practice, 60123 Ancona, Italy; 4 Department of Conservative and Endodontic Dentistry, Strasbourg University, Strasbourg, France; 5 Department of Bioengeneering and Biomaterials, INSERM UMR_S 1121, Strasbourg University, Strasbourg, France; 6 Pôle de Médecine et Chirurgie Bucco-Dentaire, Hôpital Civil, Hôpitaux Universitaire de Strasbourg, Strasbourg, France; 7 Private Practice, Hollerich Healthcare Center, Hollerich-Luxembourg, Luxembourg

**Keywords:** retreatment, calcium silicate materials, mineral deposition, endodontic material removal

## Abstract

**Objectives:**

Calcium silicate materials are widely used in endodontic treatment. Different calcium silicate percentages can be included in bioceramic sealers. The aim of this *in vitro* study was to investigate the effect of the calcium silicate percentages on mineral deposition into dentinal tubules at 7 days, 1 month, and 4 months of aging, as well as the effect of calcium silicate percentages on the quality of retreatment using two endodontic retreatment systems.

**Materials and Methods:**

Single rooted premolars were used in the present study. After the shaping and irrigation steps, the obturation was performed using high (Ceraseal “CRS”) and low (AH Plus Bioceramic “AHB”) calcium silicate percentage sealers. ReTreaty (RT) and ProTaper Universal Retreatment (PUR) were used to perform the retreatment process. The time required to achieve the apex was recorded. A digital microscope and a cone-beam computed tomography (CBCT) were used to evaluate the remaining materials after the retreatment procedure. Scanning electron microscope was used to investigate the presence of mineral deposition into dentinal tubules and the change in mineral morphology at 7 days, 1 month, and 4 months. The data was statistically analyzed using two-way analysis of variance and *t* -test.

**Results:**

Both materials (CRS and AHB) demonstrated different mineral depositions onto their surfaces after 24 hours, 1 month, and 4 months, and showed mineral depositions into dentinal tubules at 4 months. RT was faster in achieving the apex for CRS group compared to PUR ( *p* < 0.001), while no difference was found between both systems among the AHB groups. Both retreatment systems were quicker to achieve the apex in AHB compared to the CRS group (RT *p* = 0.035 and PUR *p* < 0.001). CBCT demonstrated a more precise measurement compared to the digital microscope in which the instrument and the material factors influence the removal ability at the coronal and middle thirds ( *p* < 0.05). No significant difference was found at the apical third.

**Conclusion:**

The retreatment of AHB was easier and faster than CRS. RT demonstrated higher removal ability and faster time compared to PUR. The apical third proved to be a difficult area to achieve an optimal cleaning. Calcium silicate percentages included in bioceramic sealers could play an important role in root canal retreatment. Higher percentages of calcium silicate can decrease the capacity of the retreatment process and increase the needed time to remove the materials.

## Introduction

Successful endodontic treatment consists of achieving an appropriate access cavity, good shaping, the use of proper irrigants, and an optimal three-dimensional (3D) obturation of the root canal system using gutta-percha and suitable sealers. 1 Various materials have been employed as endodontic sealers to fill the canal and entomb and eliminate bacteria found in the dentinal tubules and the root canal system. 2 These materials include epoxy resin, zinc oxide-eugenol, Guttaflow (gutta-percha in the form of a powder in the micrometer range and a sealer, both of which cure quickly together), and calcium silicate cements, each differing in physicochemical, biological, and setting reactions. 3

For many years, epoxy resin sealers have been considered the gold standard in dental practice and have been studied. 4 5 6 Recently, calcium silicate materials, also known as bioceramics (BC), have emerged as a breakthrough in dental practice due to their ability to be set in moisture conditions and their applicability in a wide range of endodontic treatments. 7 BC materials are favored for their antibacterial activity, biocompatibility, filling ability, and physicochemical properties. These advantages make this material a preferred choice in specific endodontic treatments. 7 8 9

BC sealers are typically available in two formulations. A powder–liquid format, which requires manual mixing, and a premixed mode, which comes ready to use. 7 Alterations in the mixing procedure can influence their physicochemical properties. 10 Studies have shown that changes in the powder–liquid ratio of calcium silicate cement can increase porosity and solubility. Moreover, variations in the powder–water ratio can affect pH, setting time, calcium ion release, and radiopacity. 10 Conversely, premixed BC sealers, which were introduced in 2007, eliminate the need for manual mixing, which reduces the risk of property modifications due to improper mixing procedures. 10 These premixed sealers can be directly injected into the root canal space.

Among the newly introduced calcium silicate-based sealers, AH Plus Bioceramic (AHB; Dentsply Sirona, Ballaigues, Switzerland) is a novel premixed BC sealer containing reactive tricalcium silicate with a lower percentage of calcium silicate compared to existing BC sealers. 11 CeraSeal (CRS; Meta Biomed, Cheongju, Korea) is a contemporary premixed calcium silicate-based sealer designed for endodontic treatments. It features higher percentages of tricalcium silicate as the reactive compound, providing good biocompatibility and bioactivity. This sealer is set in moist conditions, and ensures a reliable seal and promotes healing in the periapical tissues. 7

Recently, the main discussion of the use of calcium silicate materials in endodontic treatment is their potential for retreatment, which is a critical aspect often overlooked in the initial selection of endodontic materials. 12 An endodontic retreatment might be required due to reinfection, treatment failure, or the need for further intervention. 13 Calcium silicate materials, known for their bioactive nature and moisture reactivity, present unique challenges in retreatment scenarios due to their tendency to harden and set within the root canal space. 13

The retreatment of calcium silicate-based sealers requires a nuanced approach that takes into consideration the material's physicochemical and mechanical properties, setting characteristics, and interaction with the root dentin. Moreover, the inherent hardness and adhesive properties of calcium silicate sealers can pose challenges during removal. Research suggests that ultrasonic instruments and some solvents such as formic and citric acids can improve the effectiveness of calcium silicate materials in retreatment procedures. 14 15

Various dental manufacturers have developed nickel-titanium removal systems such as the ProTaper Universal Retreatment (PUR; Dentsply Sirona) system, which consists of a multiple-file system that includes three files with convex and triangular cross-sections. The D1, D2, and D3 are used to remove the filling materials from the cervical, middle, and apical thirds, respectively. 16 Another system was developed by Shenzhen Perfect Medical Instruments, which consists of five files. The first two files, Bull-Y and Skinn-Y, are used to remove the previous materials and to reach the apex, while the other three files are used to complete the shaping procedure. 17

Considering the evolving endodontic materials, understanding the impact of calcium silicate percentages in sealers on retreatment processes is crucial for advancements in endodontic care.

This study aims to evaluate the effect of two retreatment sequences on the quality of calcium silicate removal using two BC sealers. In addition, this study explores the effect of calcium silicate percentages included in endodontic sealers on crystallization and mineral deposition into dentinal tubules within 4 months of aging. The null hypothesis was that there would be no difference between the two novel calcium silicate-based sealers (AHB “low calcium silicate percentage” and CRS “high calcium silicate percentage”) in terms of mineral deposition and retreatability.

## Materials and Methods

### Materials

Two premixed calcium silicate-based sealers, AHB (Dentsply Sirona) and CRS (Meta Biomed), were used in the present study. The chemical composition and the calcium silicate percentages of each sealer are provided in Table 1 .

**Table 1:** Manufacturer, manipulation, and calcium silicate percentages of the tested materials

Materials	Company	Calcium silicate percentages	Manipulation
CeraSeal (CRS)	Meta Biomed, Cheongju, Korea	Tricalcium silicate (20–30%) and dicalcium silicate (1–10%)	Premixed
AH Plus Bioceramic (AHB)	Dentsply Sirona, Ballaigues, Switzerland	Tricalcium silicate (5–15%)	Premixed

In addition, two endodontic retreatment systems, ReTreaty (Dental Perfect, Shenzhen City, Guangdong Province, China) and PUR (Dentsply Sirona) were used.

Seventy-eight single rooted mandibular premolars with a fully developed root system and a curvature of less than 15 degrees were collected according to the Schneider technique. 18 The project was approved by the Ethics Committee at the Ministry of Higher Education in Syrian Arab Republic (3180/412/2024), and the study respected the ethical values of the Declaration of Helsinki.

### Preparation of Teeth and Retreatment Process

Sixty extracted lower premolars with no caries and endodontic treatment were selected for this study. The sample size calculation was based on a previous study 19 ; thus, 15 samples were used for each group to evaluate the remaining materials after the use of two retreatment systems. The exclusion criteria were teeth with resorption, open apex, curvatures more than 15 degrees, and cracks. The teeth crowns were sectioned using diamond disks to attain a 17-mm length and a working length (WL) of 16 mm. All the endodontic steps, including access cavity, shaping, irrigation, obturation, and retreatment, were performed by the same operator (an endodontist with 8 years of experience). The access cavity was prepared using diamond burs, then a 10 K-file (Shenzhen Perfect Medical Instruments Co. Ltd) was inserted to reach the apical foramen. All teeth were set to a WL by using MG3 GOLD (Shenzhen Perfect Medical Instruments Co. Ltd) to 25/04 at a speed of 350 revolutions per minute (rpm) and a torque of 3 Ncm. The canals were irrigated with 2 mL of 5.25% NaOCl (sodium hypochlorite, Septodont, Saint-Maur-des-Fossés, France) between each file using a 30-gauge side-vented needle (Endo-Eze, Ultradent Products, Inc.). Apical patency was checked with #10 K-file during the root canal preparation. Note that 5.25% NaOCl and 17% ethylenediaminetetraacetic acid (EDTA, Septodont) were used at the final irrigation and the activation was performed using Irriflex needles (Produits Dentaires SA, Vevey, Switzerland).

After drying with paper points, the root canals were filled through the single-cone technique (gutta-percha cone “Shenzhen Perfect Medical Instruments Co. Ltd”) using a calcium silicate-based sealer (AHB or CRS) according to the sample distribution (30 teeth for each group). Cone-beam computed tomography (CBCT) (PaX-i3D Green unit, Vatech, Hwaseong-si, Republic of Korea) was employed to verify the quality of the obturation for all filled teeth. A temporary filling material (Cavit, 3M, Germany) was used to fill the access cavities. The samples were stored in phosphate-buffered saline (PBS) for 28 days to ensure an appropriate setting of the filling materials.

### Retreatment Procedures

As mentioned, 30 teeth were filled with AHB and 30 teeth were filled with CRS. Half the number of teeth of each group ( *n* = 15) was retreated using PUR at a speed of 350 rpm and a torque of 1.5 Ncm. For the coronal third, D1 file (tip 30) was used to remove the filling materials, then the D2 file (tip 25) was used for the middle part, and then D3 (tip 20) until the WL for both sealers was reached (CRS, AHB). The other half was retreated with RT at a speed 400 rpm and a torque of 1.5 Ncm. For the coronal part, the BULL(Y) file (25/07) was employed to remove the coronal gutta-percha, and the SKINN(Y) file (25/04) was used to reach the WL. After that, the shaping file was used to completely remove the sealer. SHAP(Y)1 (20/05), then SHAP(Y)2 (25/05), and lastly SHAP(Y)3 (30/05) were used to remove the remaining materials.

A 5 mL of 5.25% NaOCl was applied between each file to remove the debris. For the final irrigation, 10 mL of 5.25% NaOCl, then 5 mL of saline, then 3 mL of 17% EDTA, and finally 5 mL of saline were applied for each canal. The retreatment was considered done when a clean irrigation fluid was observed.

### Evaluation of Remaining Materials

**Fig. 1:**
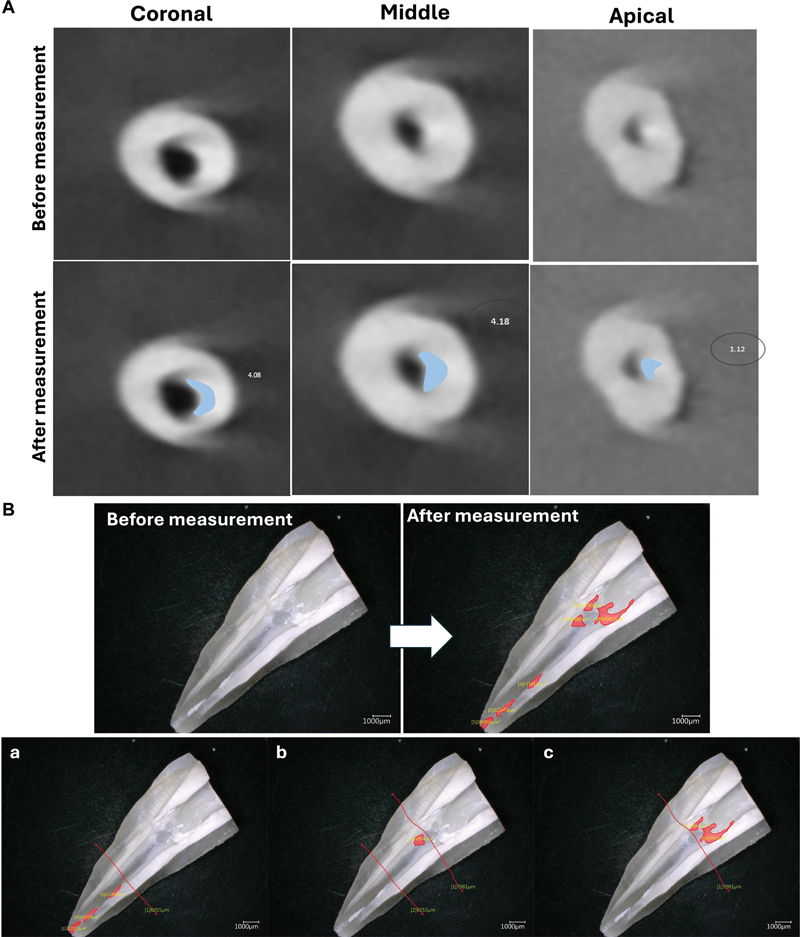
( **A** ) Cone-beam computed tomography (CBCT) images demonstrate cross-sections plans at the apical, middle, and coronal thirds. ( **B** ) Digital images of the remaining materials after root canal retreatment: Methodology of the calculation of the remaining materials using VHX-5000 Keyence. The measurement of the remaining materials in the (a) apical; (b) middle; and (c) apical.

After 28 days of immersion in PBS at 37°C, all the teeth were scanned using CBCT (PaX-i3D Green unit, Vatech). The conditions of exposition of the samples were 90 kV and 10.2 mA with a field of view of 5 × 5 cm and an isotropic resolution of 0.1 mm, with 12.57 seconds of exposure time. Each tooth was evaluated at the apical and middle thirds in cross-section plans ( Fig. 1A ). Two observers analyzed the images to obtain percentages for the remaining materials. After the CBCT, all the teeth were fractured longitudinally in the axe of the tooth in order to have a global vision of the canal. After that, a digital microscope (Keyence, Osaka, Japan) was used to observe the remaining materials in the canal at the apical and middle thirds and to precisely detect the type of the remaining materials (gutta-percha or/and sealer). The percentage of the remaining materials in the canal was calculated using VHX-5000 Keyence. The results are shown in Fig. 1B .

### Scanning Electron Microscope of Crystallization and Mineral Deposition

Teflon molds (height: 3.8 mm; diameter: 3 mm) were used in the present study to prepare cylinders of each material ( *n* = 9). After a proper setting time of 48 hours in dark containers with 95% of humidity, they were placed in PBS at 37°C for different time points ( *n* = 3): 24 hours, 1 month, and 4 months. After each immersion duration, the samples were gently rinsed with distilled water for 5 minutes and mounted on scanning electron microscope (SEM) stubs. The samples were sputter coated with gold/palladium (Technics, California, United States) and observed under SEM microscope (FEI Company, Eindhoven, The Netherlands, 10 kV). Energy dispersive X-ray analysis was performed with a working distance of 10 mm and an acquisition time of 60 seconds at 4,000× magnification. The weight percentages of chemical elements of the surfaces of the specimens of the different sealers were attained.

Furthermore, 18 single-rooted teeth were prepared as demonstrated in the “Preparation of teeth and retreatment process” section and obturated with AHB and CRS ( *n* = 9), then divided into three groups of three teeth for aging periods of: 24 hours, 1 month, and 4 months. The samples were used to evaluate the mineral depositions into dentinal tubules after the obturation of each material. Each sample was sectioned longitudinally, and the surfaces were prepared by 10 seconds of phosphoric acid, rinsing with distilled water, and then immersing in 3% NaOCl for 2 minutes. After that, the samples were dried by ethanol and mounted onto SEM stubs. After sputter coating, the samples were observed under SEM to observe the presence of mineral depositions on the dentinal tubules.

### Statistical Analysis

Sigma Plot (11.2, Systat Software, Inc., San Jose, California, United States) was used. Two-way analysis of variance (ANOVA) including multiple comparison procedures (Holm–Sidak method) was performed to investigate whether a significant difference exists between both retreatment systems and both sealers in terms of the quantity of removed/remaining materials. Moreover, *t* -test was used to compare the needed time for each system to reach the apex. The normality of data within both groups was tested using the Shapiro–Wilk test. A significance level at *α* = 0.05 was adapted.

## Results

### Mineral Depositions and Chemical Analysis

**Fig. 2:**
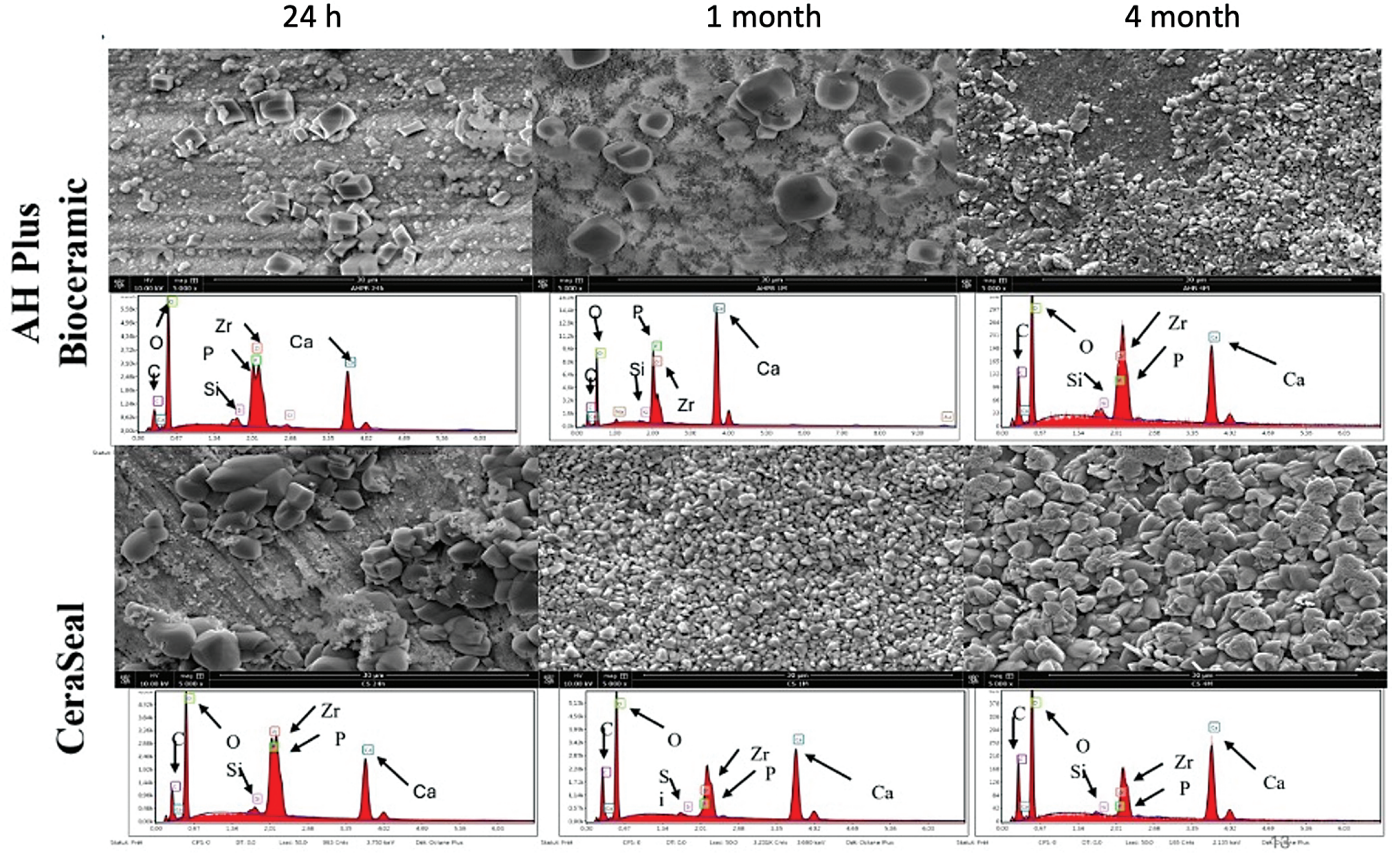
Scanning electron microscope (SEM) images demonstrated the morphological changes onto the surfaces of each material after 24 hours, 1 month, and 4 months. Energy dispersive X-ray (EDX) showed the chemical compositions of each surface after the different aging periods.

Different sizes, shapes, and quantities of crystalline were observed on both material surfaces after 24 hours, 1 month, and 4 months. After 1 and 4 months, higher qualitative number of mineral depositions was observed with CRS compared to AHB ( Fig. 2 ). Both materials demonstrated a deposition of Ca, Si, Zr, and P on their surfaces. Lower Si percentages were observed after 1 and 4 months.

### Mineral Deposition into Dentinal Tubules

**Fig. 3:**
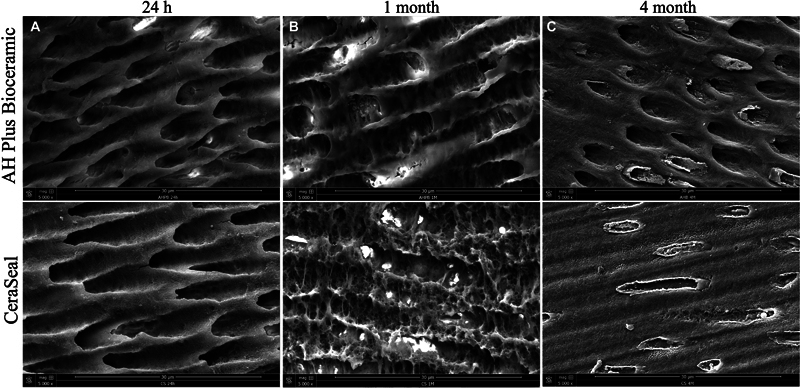
Scanning electron microscope (SEM) images demonstrated ( **A** ) mineral depositions into dentinal tubules after 4 months of aging in both groups AH Plus Bioceramic (AHB) and CeraSeal **(** CRS) and ( **B** and **C** ) dentinal tubules without the presence of mineral deposition at 24 hours and 1 month.

Both materials demonstrated mineral deposition into dentinal tubules in several zones of the root structures after 4 months ( Fig. 3A ). At 24 hours and 1 month, the samples did not demonstrate any mineralization ( Fig. 3B and C ).

### Retreatment Required Time

Statistically, less time was required to arrive at the apex with AHB compared to CRS, regardless of the retreatment system used (RT *p* = 0.035 and PUR *p* < 0.001) ( Table 2 ). Concerning the AHB group, no significant difference was observed between RT and PUR within the needed time ( *p* = 0.861). As for the CRS group, significantly less time was needed to reach the apex using the RT group compared to the PUR group ( *p* < 0.001).

**Table 2:** Mean and standard deviations of needed time to attain the apex

Materials	Test	RT	PUR	Statistical analysis
CeraSeal (CRS)	Time (min, s)	2.33 ± 0.81 ^a,A^	3.83 ± 1.36 ^b,A^	a < b
AH Plus Bioceramic (AHB)		1.65 ± 0.55 ^B^	1.48 ± 0.29 ^B^	No
Statistical analysis “ *t* -test”		A > B	A > B	

Abbreviations: PUR, ProTaper Universal Retreatment; RT, ReTreaty.

Note: Superscript small letters indicate statistically significant differences in rows and capital superscript letters indicate statistically significant differences in columns.

### Digital Microscope Remaining Materials Evaluations

Two ways ANOVA including pairwise comparison procedures (Holm–Sidak method) were used to statistically compare the remaining materials in each third after the evaluation using a digital microscope with VHX-5000 software. At the coronal third, both material and used instruments had statistically significant influence on the results. The use of RT generated fewer remaining materials after retreatment compared to PUR ( *p* = 0.018), which was statistically significantly different. Moreover, CRS demonstrated a significantly higher amount of remaining material compared to AHB ( *p* = 0.005). In contrast, at the middle third, the difference in the mean values among the different levels of materials ( *p* = 0.739) and instruments ( *p* = 0.097) were not high enough to exclude the possibility that the difference was just due to random sampling variability after allowing for the effects of differences in materials or instruments. The same observation was found at the apical third materials ( *p* = 0.849) and instruments ( *p* = 0.186) ( Table 3 ).

**Table 3:** Mean and standard deviations of remaining material percentages analyzed using a digital microscope at apical (A), middle (M), and coronal (C) thirds among the AHB and CRS groups using RT and PUR systems

Materials	Test	RT	PUR	Statistical analysis
CeraSeal (CRS) (mm ^3^ )	C ^x^	472 ± 817 ^a^	761 ± 685 ^b^	a < b
	M	337 ± 679	392 ± 466	No
	A	345 ± 258	399 ± 557	No
Ah Plus Bioceramic (AHB) (mm ^3^ )	C ^y^	158 ± 319 ^a^	407 ± 396 ^b^	a < b
	M	232 ± 347	98 ± 111	No
	A	568 ± 1111	173 ± 138	No
Statistical analysis	y < x			

Abbreviations: PUR, ProTaper Universal Retreatment; RT, ReTreaty.

Note: Superscript letters (a and b) indicate statistical significances between the group in row and (x and y) in columns.

### CBCT Microscope Remaining Materials Evaluations

Two ways ANOVA including pairwise comparison procedures (Holm–Sidak method) demonstrated that at the coronal third, both material and used instruments had a significant influence on the results. The use of RT generated significantly fewer remaining materials after retreatment compared to PUR ( *p* = 0.003), and CRS demonstrated significantly higher remaining material compared to AHB ( *p* = 0.006) ( Table 4 ). At the middle third, the same observations were obtained for the influence of instruments ( *p* = 0.003) and the materials ( *p* = 0.001). In contrast, at the apical thirds, the difference in the mean values among the different levels of materials ( *p* = 0.298) and instruments ( *p* = 0.099) was not great enough to exclude the possibility that the difference is just due to random sampling variability after allowing for the effects of differences in materials or instruments.

**Table 4:** Mean and standard deviations of remaining material percentages analyzed using a CBCT at apical (A), middle (M), and coronal (C) thirds among the AHB and CRS groups using RT and PUR systems

Materials	Test	RT	PUR	Statistical analysis
CeraSeal (CRS) (%)	C ^x^	9.5 ± 7.7 ^a^	20.1 ± 15.8 ^b^	a < b
	M ^p^	14.2 ± 9.7 ^a^	21.6 ± 13.7 ^b^	a < b
	A	15.5 ± 7.3	17.5 ± 11.9	No
Ah Plus Bioceramic (AHB) (%)	C ^y^	5.34 ± 6.05 ^a^	10.1 ± 5.37 ^b^	a < b
	M ^z^	5.07 ± 3.7 ^a^	13.2 ± 9.4 ^b^	a < b
	A	6.54 ± 3.14	18.06 ± 27.9	No
Statistical analysis	y < x – z < p			

Abbreviations: CBCT, cone-beam computed tomography; PUR, ProTaper Universal Retreatment; RT, ReTreaty.

Note: Superscript letters (a and b) indicate statistical significances between the group in row and (x, y and p, z) in columns.

## Discussion

Calcium silicate materials, including sealers and cements, are widely used in dentistry, especially in the endodontic field. Physicochemical properties of endodontic sealers, including stability over time, sealing ability, and biological properties such as antibacterial properties and biocompatibility, along with cost-effectiveness, are essential criteria influencing clinicians' decisions. 20 21 More than 51.70% of dental practitioners use these materials in their practice, 22 as these materials are known for their good biological, physicochemical, and mechanical properties. 23 Calcium silicate sealers and cements can be delivered in powder–liquid or premixed formulations; however, premixed ones demonstrated higher homogeneity and simpler use compared to the manual-mixed products. 24 Despite their strong biological effects, these materials have several negative points such as their high solubility and the difficulty of their removal from the apical thirds in retreatment cases. 25 26 27 Various papers proposed the use of different special irrigations such as formic and citric acids in order to facilitate the removal of these materials during endodontic mechanical processes. 14 15 Some dental manufacturers tried to alter the calcium silicate percentages in an attempt to facilitate the retreatment process. A previous study demonstrated that the use of a low calcium silicate sealer (AHB = 5–15%) can decrease the fracture resistance of this material compared to a standard one, which contains 40 to 50%. 28 In the present study, two sealers with two different calcium silicate percentages were used to obturate the teeth as well as to investigate their mineral depositions into dentinal tubules and their crystalline formations on their surfaces in contact with PBS at 24 hours, 1 month, and 4 months. In addition, two different retreatment systems were used to compare their efficacity in calcium silicate retreatment.

The results of the present study demonstrated that the changes in calcium silicate percentages could affect the mineral deposition shapes and quantity, as well as the ability of retreatment. Moreover, the use of different retreatment systems can influence the quality of calcium silicate removal, and the time needed to reach the apex. Therefore, the null hypothesis must be rejected ( *p* < 0.05).

SEM observations demonstrated different sizes, shapes, and numbers of mineral depositions onto each sealer after the different studied times. CRS demonstrated higher and bigger mineral depositions compared to AHB, which could be related to their different chemical compositions, especially the higher percentages of calcium silicate that are included in CRS. Lower Si percentages were observed after 1 and 4 months in both products. This could be linked to the high activity of crystalline formations, which hide the surface of the material. SEM was also used to detect the presence of mineral deposition into dentinal tubules at the different time points. As shown in the “Results” section, both materials demonstrated mineral depositions in the dentinal tubules after 4 months of incubation and contact with PBS. This observation was also reported previously for the use of mineral trioxide aggregate where the authors showed mineral deposition into dentinal tubules after 4 months of aging. 29 Therefore, calcium silicate-based materials could enhance the mineral depositions due to the liberation of calcium ions and the alkaline pH. 13 These mineral depositions can entomb the bacteria into dentinal tubules. Moreover, these structures appear to be important in the sealing of dentinal tubules and the biomineralization of the material. 29 Therefore, due to Ca ^2+^ -H ^+^ ion exchange, SiO ^−^ formation, interaction between SiO ^−^ and Ca ^2+^ charge, the formation of mineral depositions, and deposition of amorphous calcium phosphate could occur. 30

Reaching the apex in the AHB group was faster than the CRS group regardless of the retreatment system. This could be explained by the lower calcium silicate percentages, which were included in AHB compared with CRS. A previous study demonstrated that AHB had lower fracture resistance values compared to a standard calcium silicate sealer. 28 Moreover, this lower calcium silicate percentage could affect the solubility, thus explaining the high solubility detected in the previous study. 31 This high solubility could affect the bond strength to dentin, create bacterial pathways, and allow the formation of voids, which decrease the fracture resistance. 4 7 23 28

Reaching the apex in RT for CRS was significantly faster than PUR, while for the AHB group, where the sealer had lower calcium silicate percentages, there was no significant difference between RT and PUR. When AHB was used, both retreatment systems could easily reach the apex due to the low calcium silicate content, which affects the fracture resistance of this sealer. 28 In contrast, CRS has a high percentage of calcium silicate elements, and the retreatment of this product is difficult. In addition, RT was significantly faster in reaching the apex than PUR, despite PUR system having three files, compared to the five instruments available with RT. This observation could be due to the difference in tip size and the taper between both systems. RT has five files, but only two of them are used in the retreatment procedure, which helps it reach the apex faster than PUR, which has three files (coronal, middle, and apical). In addition, the other three RT files are used for reshaping the canal. The last one has a 30-tip size and 5% taper, which make the retreatment process faster compared to PUR, which has a 20-tip size. Therefore, it requires more time to remove the previous filling materials, especially in the present study the canals were prepared to 25/04.

The remaining materials in each third were measured using a digital microscope with VHX-5000 software. In the apical and middle thirds, there were no significant difference between the groups, and there was no influence of the materials or the instrument on the quality of retreatment. In contrast, at the coronal third, RT demonstrated significantly better results than PUR in the CRS groups. In all cases, the remaining materials at the coronal third could be eliminated using ultrasonic tips. 32 Therefore, the most important third is the apical one. The use of the digital microscope, as a two-dimensional (2D) microscope, could give only 2D sections and it cannot be possible to measure the thickness of the remaining materials. 33 Therefore, a 3D device was used to achieve this approach. A CBCT was used in order to have more clear measurement in 3D. 32 In the coronal and middle thirds, AHB had significantly less remaining materials than CRS, which is related with the calcium silicate percentages that decrease the fracture resistance. 28 RT showed statistically higher ability to remove the materials compared to PUR. In addition, the difference between the cross-section of both systems plays a role in some way, whereas the PUR has a convex triangle cross-section with three cutting angle that increase the engagement to the filling of root canal with less impulsion debris coronally. In contrast, the RT has two cutting angles and therefore more space to impulse the debris out and less engagement with the filling materials that improve the effectiveness.

At the apical third, both instruments, regardless of different apical sizes (D3 = 20/07 and SHAP(Y)3 = 30/05), could not eliminate totally the remaining material and there was no significant difference between their removal ability. The apical zone is a very difficult zone where the ultrasonic tips are not accessible, thus chemical actions using citric or formic acids 14 15 associated to a mechanical process could be proposed to eliminate the remaining materials in the apical third. In addition, it was reported that master apical preparation size ≥ 30 may result in an increased healing outcome in terms of clinical and radiographic evaluations 34 as well as an increase in the apical preparation size significantly enhanced root canal disinfection. 35

The primary goal of each endodontic treatment is success, and the best technique and materials should be applied to achieve and increase the success rate. However, sometimes, missing canal, calcified canal, short filling, and some other factors could influence negatively the success of the endodontic treatment, thus a retreatment should be performed. Further studies should be performed to investigate the mechanical and morphological changes in dentinal structure after long contact with calcium silicate materials and if the mineralization into dentinal tubules could increase or decrease the fracture resistance of radicular dentin. Moreover, further investigations should be performed by using a micro-CT in order to achieve higher resolution of the remaining materials compared to a CBCT. Another limitation of the digital microscope, that the preparation of the samples including sectioning, could generate debris or eliminate some remaining materials, therefore, this technique could affect the results. Moreover, the evaluation of the volume of mineral deposition after SEM analysis was performed qualitatively, thus a verified software should be used in further studies to assess this evaluation. Further studies should include several time points for the ability of retreatment of calcium silicate, especial for the few first days. In addition, only premolars were used in the present study, various anatomies such as ovals canals should be studied in further research works.

## Conclusion

Calcium silicate percentages included into BC sealers could play an important role in root canal treatment. Higher percentages of calcium silicate could decrease the capacity of retreatment process and increase the needed time to remove the materials. CRS demonstrated longer time needed to be retreated and lower removal ability compared to AHB. In addition, RT demonstrated less time needed to attain the apex and less remaining materials at the coronal and middle thirds. No influence of the retreatment instrument of the used sealer was found in the apical third among the removal ability. Further studies on AHB should investigate the solubility, setting time, and calcium releasing as well as its biological activities to evaluate the impact of calcium silicate percentages on these properties.
